# The risks of overlooking the diagnosis of secreting pituitary adenomas

**DOI:** 10.1186/s13023-016-0516-x

**Published:** 2016-10-06

**Authors:** Thierry Brue, Frederic Castinetti

**Affiliations:** 1 Hôpital de la Conception 147, Boulevard Baille, 13285 Marseille Cedex 05, France; 2 Pôle ENDO (Endocrinologie Nutrition Diabète Obésité), 147 Boulevard Baille, 13285 Marseille Cedex 5, France

**Keywords:** Acromegaly, Cushing’s disease, Pituitary neoplasms, Prolactinoma, Hyperthyroidism, Pituitary hormones

## Abstract

Secreting pituitary adenomas that cause acromegaly and Cushing’s disease, as well as prolactinomas and thyrotroph adenomas, are uncommon, usually benign, slow-growing tumours. The rarity of these conditions means that their diagnosis is not familiar to most non-specialist physicians. Consequently, pituitary adenomas may be overlooked and remain untreated, and affected individuals may develop serious comorbidities that reduce their quality of life and life expectancy. Because many signs and symptoms of pituitary adenomas overlap with those of other, more common disorders, general practitioners and non-endocrinology specialists need to be aware of the “red flags” suggestive of these conditions. A long duration of active disease in patients with secreting pituitary adenomas is associated with an increased risk of comorbidities and reduced quality of life. Appropriate treatment can lead to disease remission, and, although some symptoms may persist in some patients, treatment usually reduces the incidence and severity of comorbidities and improves quality of life. Therefore, correct, early diagnosis and characterization of a pituitary adenoma is crucial for patients, to trigger timely, appropriate treatment and to optimize outcome. This article provides an overview of the epidemiology of hormonal syndromes associated with pituitary adenomas, discusses the difficulties of and considerations for their diagnosis, and reviews the comorbidities that may develop, but can be prevented, by accurate diagnosis and appropriate treatment. We hope this review will help general practitioners and non-endocrinology specialists to suspect secreting pituitary adenomas and refer patients to an endocrinologist for confirmation of the diagnosis and treatment.

## Background

Pituitary adenomas are usually benign, slow-growing tumours; those that secrete an active hormone are known as “hormonally active” or “functional tumours” and, unlike the so-called “non-functioning” pituitary adenomas, lead to diseases of excessive hormone secretion. The secreting pituitary adenomas include those that cause acromegaly and Cushing’s disease as well as prolactinomas and thyrotroph adenomas. Although gonadotroph adenomas may exceptionally trigger symptoms related to gonadotropin hypersecretion, they account for the vast majority of non-functioning pituitary adenomas and therefore will not be discussed in the present review [1, 2].

All types of pituitary adenomas may compress surrounding structures, primarily the normal pituitary gland and optic pathways, thus causing symptoms of hypopituitarism, headaches, and visual disturbance [3, 4]. Despite their benign nature, pituitary adenomas may invade the adjacent cavernous sinus, a feature making anatomical and histological invasion a key prognostic factor for these tumours [5–8] and a basis for their classification [9]. However, the present article will focus on the manifold consequences of pituitary hormone overproduction by the different types of hormonally active adenomas. Acromegaly is caused by hypersecretion of growth hormone (GH), which leads to increased levels of circulating insulin-like growth factor 1 (IGF-1) [10], and Cushing’s disease arises from chronic hypercortisolism associated with oversecretion of adrenocorticotropic hormone (ACTH) [11]. In patients with prolactinomas, excess prolactin can lead to gonadal dysfunction due to decreased levels of oestrogen in women and testosterone in men, and to oligo-amenorrhoea and galactorrhoea in premenopausal women. Prolactinomas in any adult patient can cause gonadal dysfunction and infertility. Finally, thyrotroph adenomas can lead to hyperthyroidism with inappropriately normal or increased thyroid-stimulating hormone (TSH) levels [4].

Secreting pituitary adenomas clearly meet the European definition of rare diseases, because they affect fewer than 1 in 2,000 individuals. Therefore most non-specialist physicians have limited experience of these conditions. In addition, many signs and symptoms overlap with those of other, more common, disorders and may be overlooked. General practitioners (GPs), to whom patients may present, and non-endocrinology specialists, to whom patients may be initially referred, therefore need to be aware of the “red flags” suggestive of a pituitary adenoma when making a diagnosis. Additionally, the techniques and assays used for biochemical diagnosis may present difficulties in routine use in the clinical setting. These factors combined mean that diagnosis of these disease states, particularly acromegaly and Cushing’s disease, may be delayed.

Generally, in patients with pituitary adenomas, a long duration of active disease is associated with an increased risk of comorbidities and decreased quality of life, so treatment should be started as soon as possible to prevent or at least limit deleterious effects of hormone excess. Compared with no treatment, appropriate treatment (surgical removal of the tumour and pharmacological management, if needed, followed by radiotherapy) can lead to disease remission, improved quality of life, decreased incidence and severity of comorbidities, and lower mortality [3, 4, 10, 12, 13]. A correct and early diagnosis is therefore crucial for patients, to trigger appropriately early treatment and to optimize outcomes.

To assist GPs and non-endocrinology specialists in recognizing and managing secreting pituitary adenomas, this review provides an overview of the epidemiology of these conditions, discusses the difficulties of their diagnosis, and examines associated comorbidities that may be prevented by accurate, early diagnosis, and appropriate treatment.

## Difficulties with diagnosis of secreting pituitary adenomas and consequences of delayed diagnosis

Historically, secreting pituitary adenomas were considered to be particularly rare. In general, however, autopsy and radiological studies reveal pituitary adenomas in 15–20 % of normal subjects [14, 15]. As diagnostic techniques have progressed, in particular the availability of specific, sensitive biochemical assays, more accurate epidemiological data on these tumours are becoming available [14–16].

Differential diagnosis of secreting pituitary adenomas is based on biochemical evaluation and imaging to assess the extent of hormone hypersecretion and to identify the location and size of tumours. Conditions associated with pituitary adenomas present in non-specific ways at an early stage, making diagnosis from clinical signs and symptoms at presentation challenging, and resulting in the possibility of diagnostic delay. Diagnosis may be further complicated and delayed by subclinical disease, the slow manifestation of symptoms in these chronic conditions, and the extensive overlap of signs and symptoms with those of other diseases [4, 13, 17–19], as discussed below. Patients are often referred to a specialist because of a certain symptom (for instance to a dermatologist for purple striae, to a rheumatologist for joint pain, to a psychiatrist for mood changes, etc.), but the non-endocrinology specialist may not consider features beyond their specialty or be aware of a complete history of the disease, and this factor may also contribute to a delay in diagnosis of a secreting pituitary adenoma.

Because changes in appearance due to acromegaly and Cushing’s disease generally occur slowly, they are often overlooked by family members, friends, GPs, and the patients themselves. There is increasing support for the development of automated diagnostic tools that would potentially aid the diagnostic process, including face-recognition software to identify the characteristic physical changes associated with these conditions [20–22], or 3-dimensional cephalometry [23]. However, such approaches have to date been applied only to limited numbers of subjects in pioneering studies.

The consequences of delayed diagnosis are an increased number and severity of complications, delayed intervention, a reduced quality of life for patients, and an increased risk of mortality. Therefore, early diagnosis is important. It allows timely intervention and the initiation of appropriate treatment, thus limiting the complications and sequelae of these conditions and allowing the possibility of remission in some cases. However, as pituitary disorders are rare and the costs of tests are relatively high, routine screening may be difficult to justify in many countries.

Finally, it must be kept in mind that pituitary adenomas may rarely be part of a type 1 multiple endocrine neoplasia (MEN-1) syndrome that also includes primary hyperparathyroidism and gastroenteropancreatic endocrine tumours. In such a setting, overlooking a diagnosis of pituitary adenoma may be particularly harmful [24].

## Acromegaly

The reported incidence of GH-secreting adenomas that cause acromegaly is 1–4 per million per year [25–30], with a prevalence of 27–97 per million [25–27, 30–34]. However, these figures may be underestimates of the true number of patients with acromegaly, and some studies suggest that the condition is more common: an incidence of 11 cases per million per year was recently found from an analysis of a large US health-plan database [34], and a German study reported a prevalence of 1,034 per million [35]. It is currently not known who is at risk of a GH-secreting adenoma. Acromegaly is slightly more common in females than in males, but men tend to be diagnosed with the disorder earlier in life than women, usually before 45 years of age [26, 28, 31, 36–41].

**Fig. 1:**
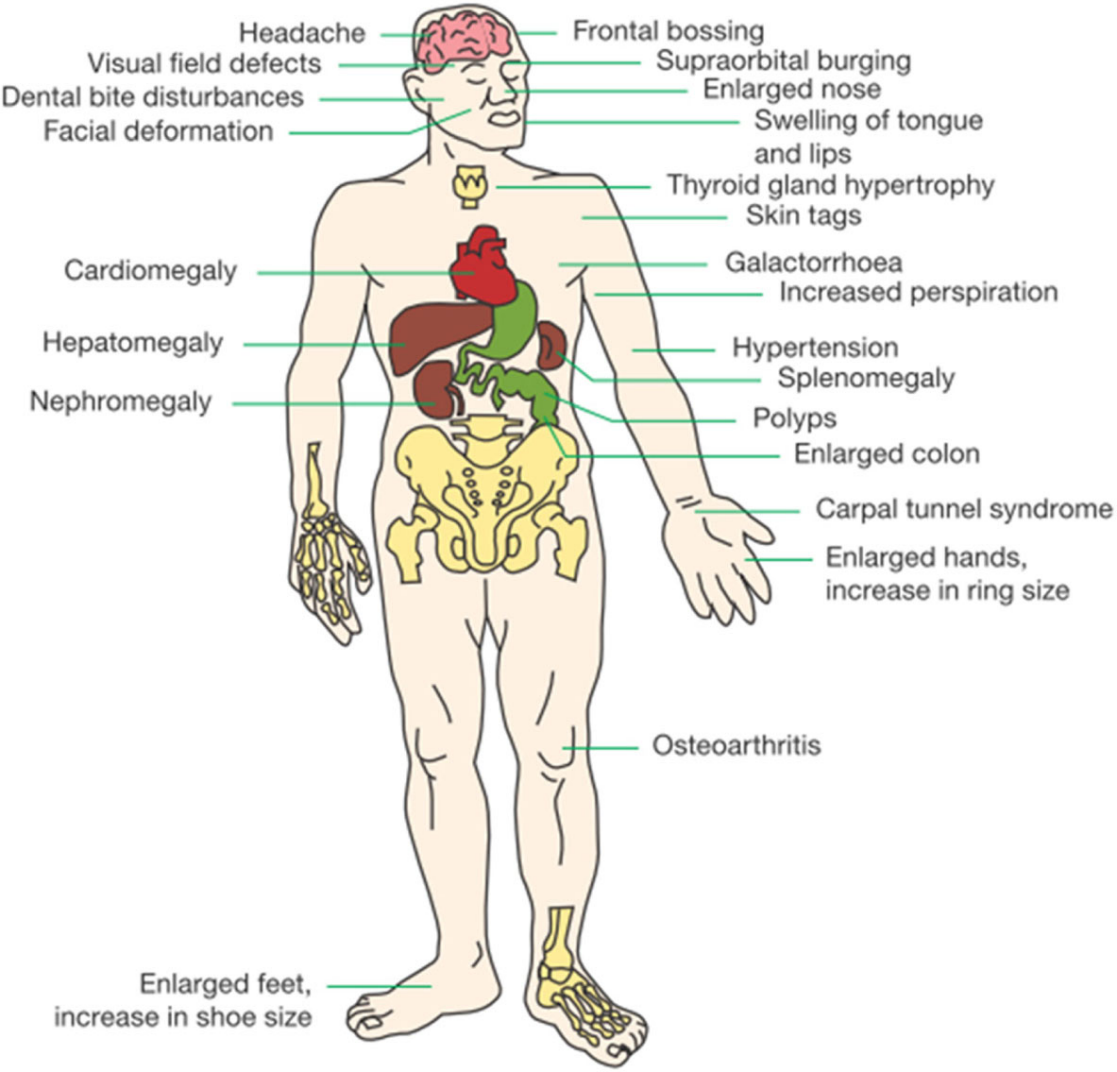
Typical features of acromegaly

**Fig. 2:**
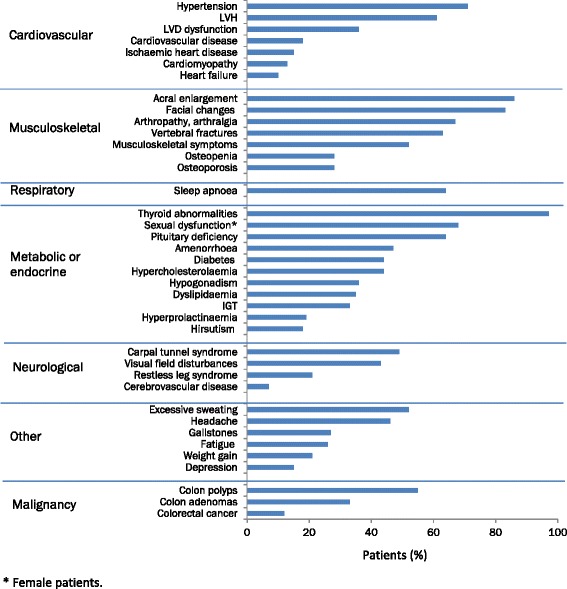
Maximal reported rates of the most common manifestations of acromegaly [25, 26, 31, 33, 36, 56–58, 77, 80, 85, 86, 97, 222–234]. *IGT* impaired glucose tolerance; *LVD* left ventricular diastolic; *LVH* left ventricular hypertrophy

Acromegaly is characterized by slowly progressive somatic disfigurement and systemic manifestations as depicted in Fig. 1, at the maximal reported rates shown in Fig. 2. For differential diagnosis, the most recent joint United States Endocrine Society and European Society of Endocrinology Clinical Practice Guideline for acromegaly recommends measurement of IGF-1 levels in patients with typical clinical manifestations of acromegaly, especially those with acral and facial features (Fig. 1) [10]. As a result of the overlap of symptoms with those of other conditions such as sleep apnoea syndrome, type 2 diabetes mellitus, arthritis, carpal tunnel syndrome, hyperhidrosis, hypertension, and cardiac disease (arrhythmias, left ventricular hypertrophy, and diastolic dysfunction), IGF-1 levels should be tested in patients without the typical features of acromegaly but who have symptoms of these conditions [10, 42, 43]. In a case of suspected acromegaly, an elevated IGF-1 level and a failure to suppress GH below 1 ng/mL during an oral glucose tolerance test (OGTT) confirm the diagnosis [10, 44].

### Difficulties with diagnosis

The slow manifestation of symptoms means that there is an average delay in diagnosis of acromegaly of 6–7 years after the first appearance of symptoms; in some patients, it may take as long as 35 years [26, 33, 41, 45]. Such a delay is partially related to the fact that slowly progressing changes in physical features may remain unnoticed by patients and the people around them until the first manifestation of complications.

It is important to be aware of a number of facts related to interpretation of test results that may complicate a diagnosis of acromegaly. Difficulties in interpreting GH level are related to the pulsatility of GH secretion, which is absent in patients with acromegaly. Increasing age, female gender, and obesity can be associated with abnormal GH suppression in response to OGTT [10]. A random GH >30 ng/mL can be seen in patients without acromegaly.

Because IGF-1 levels decrease with age after adolescence [46], they must be assessed in relation to age- and gender-appropriate normal values for the specific assay used [10]. Falsely positive diagnoses may be made in late-stage adolescent patients or during pregnancy [10]. Patients taking oral oestrogens may have low IGF-1 levels. Also, patients with hepatic or renal failure, hypothyroidism, malnutrition, severe infection, hepatic disease, or poorly controlled diabetes mellitus may have abnormal IGF-1 levels compared with healthy individuals [47–49]. In addition, because variability between GH and IGF-1 assays used at different laboratories is significant, and standardization of assays is lacking, correct interpretation of results requires knowledge of the specific assay used [10, 44, 50, 51].

A challenging diagnostic issue presents in patients with diabetes mellitus, because these patients can have an abnormal IGF-1 level or an abnormal response to OGTT (or both) [10, 52]. In diabetic patients, diagnosis is usually based on readouts from multi-sample day curves for GH, and GH values >1 ng/mL are considered abnormal. Re-evaluation by a specialized endocrinologist is highly recommended in these patients.

There is still an unmet need for validated symptom-scoring tools aiding recognition of patients with acromegaly, and research is providing some tools that may become clinically useful in due course [53–55]. The ACROSCORE is a 14-point scoring system based on the cardinal symptoms and signs of acromegaly and developed for the clinical screening of acromegaly [55]. Although still not validated, the ACROSCORE might become an easy-to-use tool to diagnose acromegaly early in the disease course, thus allowing patients with acromegaly to be distinguished from those in whom acromegaly has been ruled out.

Other tools in development combine biochemical and clinical parameters to measure disease activity, and they could be useful not only for diagnosis of acromegaly, but also for evaluating the effects of treatment [53, 54]. The SAGIT instrument is a comprehensive clinician-reported outcome tool to assess the key features of acromegaly and thus assist endocrinologists managing acromegaly in practice, with promising results from a pilot study [53]. SAGIT combines signs and symptoms, associated comorbidities, GH levels, IGF-1 levels, and tumour profile. Finally, ACRODAT is a decision algorithm based on IGF-1 level (SD score), tumour status (change on magnetic resonance imaging), comorbidities (number and severity), signs and symptoms (Patient Acromegaly Symptom Questionnaire score), and health-related quality of life (scored on a disease-specific measure) [54]. In a modelling exercise performed for this score, biochemical and tumour statuses were shown to be the primary predictors of disease activity [54].

### Consequences of delayed diagnosis

Earlier diagnosis and treatment, as well as appropriate follow-up, may potentially limit or avoid the life-long consequences of uncontrolled disease and reduce mortality risk. On the other hand, late diagnosis and therefore long-term exposure to GH and IGF-1 excess may result in comorbidities that are difficult to manage and, in some cases, may persist even after biochemical control is achieved. The most serious long-term consequences of untreated acromegaly are hypertension, cardiovascular disease, diabetes, arthropathies, and obstructive sleep apnoea [25, 29, 31, 56–60] and patients have significant deterioration in quality of life [61].

Hypertension and diabetes are very important risk factors for cardiovascular disease in patients with acromegaly, and approximately half of patients are at intermediate or high risk of coronary artery disease [62]. Patients with an estimated disease duration of longer than 10 years have a threefold higher relative risk of cardiac complications than do patients with an estimated disease duration of 5 years or less [63]. In normal health, GH and IGF-1 have a regulatory role in the cardiovascular system, and patients with elevated GH and IGF-1 levels therefore often demonstrate both structural and functional abnormalities and diastolic dysfunction, as well as abnormalities in the vascular system. Early symptoms of acromegaly-associated cardiomyopathy include cardiac hypertrophy, an elevated heart rate, and increased systolic dysfunction [42, 59, 60, 62, 63]. If left untreated, these initial symptoms may develop into more pronounced hypertrophy, diastolic dysfunction, and systolic insufficiency during exercise [59]. The standardized mortality ratio for patients with acromegaly ranges from 1.1 to 3.2 in different countries [25, 26, 36, 37, 64–66], with the main causes of death being cardiovascular disease, cerebrovascular disorders, and respiratory disorders [28, 36, 37, 65, 67, 68].

Early diagnosis and early initiation of treatment in acromegalic patients can prevent the progression of cardiovascular disease and reduce the risk of premature death [69]. However, it is still unknown for how long cardiovascular comorbidities remain reversible. Although treatment to reduce GH and IGF-1 levels can decrease risk of cardiac hypertrophy and arrhythmias, as well as improve diastolic function [70], improvements in systolic function and response to exercise depend mostly on disease duration and the presence of hypertension and diabetes [63]. Coronary artery calcifications correlate with disease duration [71], but the clinical impact of treatment on coronary artery disease remains unknown. Hypertension is the main contributor to increased mortality in acromegaly and, unlike hyperglycaemia (which resolves in the majority of patients in remission), it persists in most cases after biochemical control is achieved [72, 73]. With regard to hyperglycaemia, GH excess contributes to the development of insulin resistance [74] and endothelial dysfunction [75]. Cardiovascular risk factors for hyperglycaemia, such as glucose homeostasis alterations, may also be present in acromegalic patients despite long-lasting GH/IGF-1 control [76].

Joint problems, such as arthralgia, are common symptoms, occurring in at least half of patients with acromegaly [25, 77]. Although reduction in joint thickness upon disease control has been observed, some joint problems may persist despite treatment for acromegaly [69, 78, 79]. Due to its mechanism, arthropathy may be reversed with early treatment for acromegaly, but this is less likely if the disease has been left untreated for several years [80, 81]. Patients with acromegaly are at increased risk of vertebral fractures, but the impact of acromegaly on bone mineral density (BMD) is still unclear [82–84]. Fracture risk is significantly associated with the duration of uncontrolled disease [83, 85]. Although BMD has been shown to improve upon biochemical control, risk of vertebral fractures persists in some patients [85, 86].

In addition to physical impairment, sleep apnoea is more common and more severe in patients with active acromegaly than in those with controlled disease [87–89]. The apnoea–hypopnoea index and tongue volume have been shown to be reduced on normalization of IGF-1 in treated patients [89, 90].

Patients with acromegaly also display impairment of cognitive performance, particularly in memory tests [58, 91], and increased anxiety-related personality traits [56]. A longer duration of uncontrolled acromegaly may be associated with neurocognitive complications of greater severity. Conversely, a longer duration of postoperative biochemical remission of acromegaly is associated with a better neurocognitive state [58]. Improvements in cognition and mental health upon control of acromegaly appear to be only partial: memory recall, concentration, learning capability, and accuracy remain impaired in patients with controlled disease compared with healthy control subjects [58].

Several studies have suggested an increased risk of cancer (including those originating from intestines, brain, breast, thyroid, uterus, prostate, kidney, and skin) in patients with acromegaly [92–95]. However, in the most recent analysis of 446 patients from the German Acromegaly Registry, the overall cancer rate was slightly lower than that in the general population and not significantly higher for colorectal, breast, thyroid, prostate, and lung cancers [96]. Although IGF-1 has been shown to play a role in the development of cancerous changes in thyroid cells, an expected association of thyroid cancer with acromegaly remains controversial. Although some studies demonstrated thyroid cancer in 4.7–5.6 % of patients with acromegaly [92, 97, 98], a rate slightly higher than that in the general population, others found no increase in the prevalence of thyroid cancer in acromegalic patients compared with the general population [96, 99].

In summary, prevention of development of comorbidities associated with GH or IGF-1 excess in patients with acromegaly through early diagnosis and treatment is of great importance. The “red flags” that should trigger further investigations for acromegaly include: a long duration of signs and symptoms such as arthralgia and sleep apnoea; early onset of cardiovascular disease, impaired glucose and lipid metabolism, and osteoporosis and vertebral fractures; persistence of symptoms such as hypertension, impaired glucose metabolism, and arthralgia despite treatment; atypical diabetes; and bilateral carpal tunnel syndrome. Early intervention may limit the development of deleterious consequences of GH or IGF-1 excess.

## Cushing’s disease

The incidence of Cushing’s disease is estimated at 1.2–2.4 per million per year [100, 101], with a prevalence of 29.1 per million population [100]. Like acromegaly, Cushing’s disease is more common in females than in males, with a reported male-to-female ratio between 1:1.5 and 1:15 [100–115]. As with acromegaly, the true number of patients with Cushing’s disease may be underestimated. This is because Cushing’s disease is also found in people who are initially diagnosed with pituitary incidentalomas [116] and may also be unrecognised in individuals diagnosed with diabetes, hypertension, or depression [117–120]. For example, it has been reported that 9 % of patients with type 2 diabetes also fulfil the criteria for subclinical Cushing’s disease [121].

**Fig. 3:**
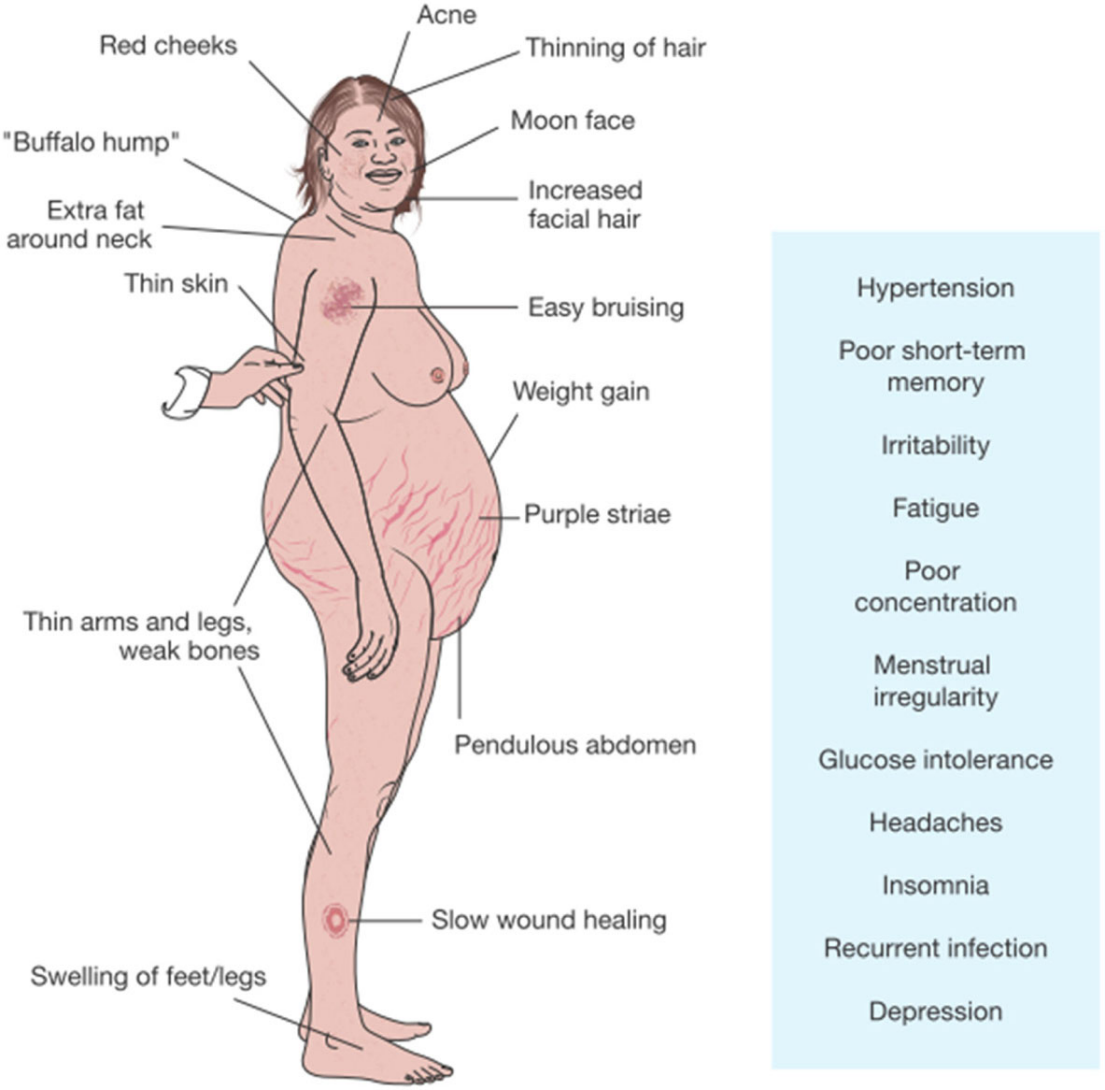
Signs and symptoms of Cushing’s disease

**Fig. 4:**
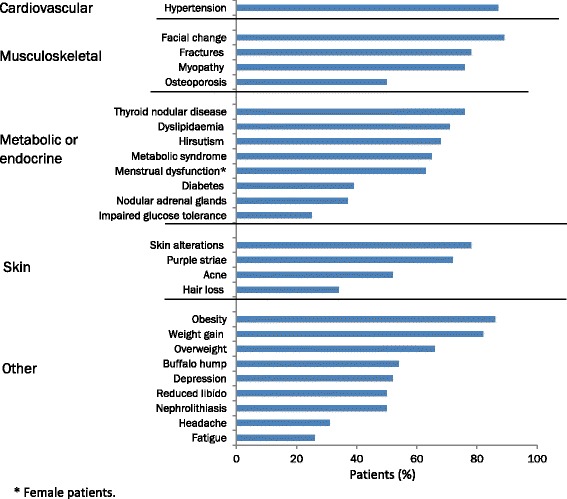
Maximal reported rates of the most common signs, symptoms, and comorbidities in patients with Cushing’s disease [100, 103, 104, 111, 112, 114, 146, 147, 235–237]

A diagnosis of Cushing’s syndrome is the usual first step for a diagnosis of Cushing’s disease and can be made once non-pituitary causes of hypercortisolism have been excluded. Detection relies first on clinical suspicion followed by biochemical confirmation [122]. The signs and symptoms of Cushing’s disease are widespread (Fig. 3), and some symptoms and complications are more frequent in males than in females. These include purple striae, muscle atrophy, osteoporosis, and nephrolithiasis [111]. The most common manifestations of Cushing’s disease are cardiovascular, metabolic, or endocrine disorders, central obesity, and dermatological features (for instance easy bruising, red face) [123, 124]; the relative incidence of the various manifestations of Cushing’s disease are summarized in Fig. 4. A definitive diagnosis of hypercortisolism can be established by repeat measurements of an elevated 24-h urinary free cortisol (UFC) concentration. Alternatively, late-night salivary cortisol levels support the diagnosis if there is a loss of the circadian rhythm of cortisol levels. Endogenous hypercortisolism is confirmed if there is a lack of cortisol suppression (below an adequate threshold, usually 50 nmol/L) after a low-dose (1 mg) overnight dexamethasone suppression test [125].

### Difficulties with diagnosis

The clinical presentation of Cushing’s syndrome or disease varies depending on the severity and duration of hypercortisolism [122]. In severe, overt hypercortisolism, the signs and symptoms (e.g. proximal muscle weakness, increased abdominal or facial fat, wasting of extremities, and wide purple striae) can easily be attributed to Cushing’s disease, but in many patients, not all the symptoms characteristic of Cushing’s disease are present, and patients with subclinical cortisol secretion or cyclic hypercortisolism may not present in a classical way [126]. Common manifestations are central weight gain, hypertension, and changes in memory, mood, and concentration. As with acromegaly, the overlap of many of the signs and symptoms of this disease with those of other conditions (such as obesity, metabolic syndrome, diabetes mellitus, hypertension, and depression) contribute to delayed diagnosis of typically 2 to 6 years after the first appearance of symptoms [19, 103, 127].

Difficulties with diagnosis may also relate to the interpretation of biochemical tests. Normal cortisol levels fluctuate in a circadian pattern; therefore, multiple tests are required to reduce the risk of false-positive or false-negative results [128, 129]. In addition, some patients (with pseudo-Cushing’s syndrome) may have an elevated UFC or abnormal response to dexamethasone (or both) and some symptoms suggestive of Cushing’s syndrome despite absence of the disease [130]. Pseudo-Cushing’s syndrome can be associated with chronic alcohol consumption, depression, severe obesity, and chronic stress [131].

Additionally, use of biochemical tests for Cushing’s disease in routine clinical practice may not be optimal, because multiple protocols and different cut-off criteria often exist for each assay [132]. Therefore expertise is needed to conduct the tests and interpret the results [129, 133].

Furthermore, many steroid medications (glucocorticoids, inhaled and topical corticosteroids, oral oestrogens) and treatment combinations including ritonavir affect cortisol levels; in some cases, this can lead to symptoms of hypercortisolism as well as affect the diagnostic utility of the results [125, 134–141].

### Consequences of delayed diagnosis

Overproduction of ACTH in patients with pituitary adenomas leads to hyperstimulation of the adrenal glands and a chronic excess of cortisol, with deleterious effects on most tissues of the body (Fig. 4), negative effects on the patient’s quality of life [13, 61, 142], and, if untreated, increased mortality [143, 144]. Mortality rates are up to 9 times higher in patients with untreated Cushing’s disease than in the general population [101, 107, 110, 115, 145]. The main complications of untreated Cushing’s disease include: hypertension; metabolic diseases such as impaired glucose tolerance, diabetes, and metabolic syndrome; myopathy; and bone-related complications such as osteoporosis and fractures [100, 104, 111, 112, 114, 116, 146–148].

Cure of the disease or at least control of hypercortisolism does not necessarily mean disappearance of comorbidities. Irreversibility of some of the complications is likely to be caused by a prolonged exposure to high cortisol levels due to the delay in diagnosis. This situation clearly emphasizes the need for greater awareness among physicians of the main “red flags” that should trigger testing for chronic hypercortisolism – uncontrolled hypertension possibly associated with hypokalaemia, atypical acquired diabetes mellitus, osteoporosis with or without vertebral fractures, hyperandrogenism and overweight (in women with a centripetal obesity), or thin skin – in patients who are younger than expected for these conditions [13].

Many cardiovascular risk factors, including hypertension, diabetes, obesity, and dyslipidaemia, are improved upon resolution of hypercortisolism, but an increased cardiovascular risk may persist and manifest in the long term [149–153]. Patients with Cushing’s disease have severe atherosclerotic damage; persistence of metabolic syndrome, vascular damage, and atherosclerotic plaques after normalization of cortisol levels contributes to a high cardiovascular risk despite treatment [150, 154]. Up to 2 % of patients with hypercortisolism die as a consequence of a thromboembolic event [155]. Factors contributing to the increased thromboembolic risk include a long duration of uncontrolled hypercortisolism, glucocorticoid-induced hypercoagulability, and obesity [112, 154]. This risk is thought to be already present 1–2 years before diagnosis of Cushing’s disease and may remain for months after surgery [113]. However, other studies report that 6 months after disease control, thromboembolic risk returns to the degree of risk seen in healthy individuals [156].

In general, the level of hypercortisolism correlates with the presence of impaired glucose tolerance, impaired insulin resistance, and diabetes [151]. Even 5 years after remission of Cushing’s syndrome, impaired glucose tolerance may persist [149]. Chronic hypercortisolism results in changes in body fat deposition and increased abdominal adiposity, with related metabolic consequences. Additionally, effects of excess cortisol in the brain can influence eating behaviour, with further contribution to the obese phenotype of patients with Cushing’s disease [157]. If the disease is controlled, significant reductions in total, abdominal visceral, subcutaneous, and bone marrow adipose tissue can be achieved, but most patients remain overweight or obese in the long term and remain at risk of cardiovascular disease [158, 159]. Dyslipidaemia tends to improve with correction of hypercortisolism, but complete normalization of lipid levels is usually not achieved, even after long-term remission [149, 150].

There are no specific studies on or guidelines for the management of cardiovascular risk factors in patients with Cushing’s disease. Nevertheless, based on the fact that these patients are usually at high or very high cardiovascular risk, standard practice should be applied to managing any cardiovascular risk factors that manifest in patients. Follow-up is recommended in the active phase of the disease and in the long term, because of the possible persistence of these risk factors after treatment-induced remission [160, 161]. The patient’s cardiovascular risk profile should be evaluated at least yearly. Hypertension, hyperglycaemia, and dyslipidaemia should be adequately treated, and particular attention should be paid to patients in remission for whom a more aggressive approach is recommended [160, 161].

Prolonged exposure to cortisol excess is detrimental to bone: reduced BMD and increased risk of osteoporosis and fractures can result [162, 163]. A study of a large consecutive cohort of 104 patients with Cushing’s syndrome demonstrated that the increased risk of fracture was confined to the last 2 years before diagnosis and the start of therapy; it reverted to normal after diagnosis and treatment [164]. This observation further supports the importance of prompt and accurate diagnosis of Cushing’s disease to prevent the effects of hypercortisolism on bone structure.

In addition, cortisol excess negatively affects the structure and function of brain tissue. Patients with active Cushing’s syndrome have a smaller hippocampus, enlarged ventricles, cerebral atrophy, and altered neurochemical concentrations and functional activity [165]. Some studies point to alterations in brain activity related to symptoms of depression and emotional memory in patients with hypercortisolism [166]. After treatment and abrogation of cortisol excess, when the patient is in long-term remission, structural and neurochemical alterations in brain tissue improve and correlate with improvements in clinical and behavioural outcomes. However, abnormalities in grey and white matter are not completely reversible and are associated with persistent psychological symptoms and impairments in cognitive functioning [165, 167].

## Prolactinomas

Prolactinomas remain the most common secreting pituitary tumours [168, 169], accounting for 40–66 % of clinically relevant cases [3, 170, 171]. A recently published Swedish study by Tjörnstrand et al. reported a standardized incidence rate (SIR) of 1.6 per 100,000 adult patients diagnosed with pituitary adenomas in one county between 2001 and 2011 (accounting for 32 % of all pituitary adenomas) [172]. Finnish data support these findings: a SIR of 2.2 per 100,000 (51 % of all pituitary adenomas) has been reported [173]. Prolactinomas have an estimated prevalence of approximately 35–50 per 100,000 inhabitants [16, 33, 174] and occur most frequently in women aged 20–50 years, with a female-to-male ratio of approximately 10:1 [175].

The effects of hyperprolactinaemia in adult patients commonly include hypogonadism, infertility, sexual dysfunction, low BMD, and effects on the mammary glands (gynaecomastia, galactorrhoea) [176–178]. During adolescence, delayed onset of puberty, oligo-amenorrhoea, and galactorrhoea may be seen in girls, and boys may have delayed pubertal development and hypogonadism [3]. “Red flags” that should trigger suspicion of prolactinoma are amenorrhoea or irregular menses associated with (inconstant) galactorrhoea, and sexual dysfunction in males.

Diagnosis of hyperprolactinaemia and identification of its cause can be based on medical history, physical examination, clinical features, serum prolactin levels, biological investigations, and imaging of the pituitary region [179, 180]. The current Clinical Practice Guideline from the Endocrine Society for the diagnosis of hyperprolactinaemia recommends a single measurement of serum prolactin, with diagnosis confirmed by a level above the upper limit of normal. Nevertheless, to account for possible prolactin pulsatility, multiple sampling (at 15- to 20-minute intervals) may be useful in confirmation of diagnosis of hyperprolactinaemia [3]. Assay-specific normal values for prolactin are higher in women than in men and generally lower than 25 μg/L [3]. Prolactin levels higher than 500 μg/L are diagnostic of macroprolactinomas [181].

In contrast with the available biochemical assays for diagnosis of acromegaly and Cushing’s disease, those for prolactinoma are roughly comparable, and assessment is usually uncomplicated in the clinical setting [3]. However, in patients with very large prolactinomas (>3 cm), prolactin assays may falsely provide a lower-than-actual prolactin level because of antibody saturation. Further dilution of the sample is needed to avoid this potential trap [182].

### Considerations for accurate diagnosis

The clinical presentation of prolactinoma is gender-specific. Women typically seek medical consultation because of the classic amenorrhoea–galactorrhoea syndrome associated with the condition, whereas men present with more general symptoms such as headache, gynaecomastia, impotence, and reduced libido [171]. Although galactorrhoea is the most characteristic manifestation of hyperprolactinaemia, it may not be present or may only manifest intermittently [183]. Up to 50 % of women with galactorrhoea have normal prolactin levels [184], but amenorrhoea associated with galactorrhoea strongly suggests hyperprolactinaemia [185].

Hyperprolactinaemia can be caused by a number of different conditions, which should be considered and ruled out to make a differential diagnosis. Some patients with non-functioning pituitary adenomas have hyperprolactinaemia resulting from compression of the pituitary stalk and are at risk of misdiagnosis [186]. Patients with acromegaly may occasionally present with markedly elevated levels of prolactin in cases of GH- or prolactin-secreting adenomas [187], and hyperprolactinaemia may occur in a subset of patients with primary hypothyroidism [188, 189].

With regard to other conditions, renal insufficiency can lead to moderate hyperprolactinaemia [190, 191], and there are exceptional cases of non-pituitary tumours secreting prolactin, for instance renal cell carcinoma, gonadoblastoma, cervical carcinoma, non-Hodgkin lymphoma, and colorectal adenocarcinoma [192–196]. In generally healthy subjects, pregnancy, breastfeeding, stress, exercise, and sleep can cause prolactin elevation [197]. Iatrogenic hyperprolactinaemia can also occur (Table 1). For instance, risperidone and metoclopramide medication can lead to prolactin levels above 200 μg/L [198, 199]. Medication-related hyperprolactinaemia has been reported at 31 % in patients treated with neuroleptics, 28 % in those treated with neuroleptic-like drugs, 26 % in patients treated with antidepressants, and 5 % in patients taking H2-receptor antagonists [200]. In such cases of drug-induced hyperprolactinaemia, prolactin elevation is usually mild but can be highly variable [201].

**Table 1:** Pharmacological causes of hyperprolactinaemia [182]

Pharmacological group	Drugs
Antipsychotics	Typical: phenothiazines, butirophenones, thyoxanthenes Atypical: risperidone, molindone, amisulpride, quetiapine, olanzapine
Antidepressants	Tricyclics: amitriptyline, desipramine, clomipramine MAO inhibitors: pargyline, clorgyline SSRIs: fluoxetine, citalopram, paroxetine
Antihypertensive drugs	Verapamil, α-methyldopa, reserpine, labetolol
Anticonvulsants	Phenytoin
Prokinetics	Metoclopramide, domperidone
Hormonal preparations	Oestrogen, danazol
H2-blockers	Cimetidine, ranitidine
Controlled substances	Opiates, methadone, morphine, apomorphine, heroin, cocaine, marijuana
Other	Anaesthetics, sibutramine, alcohol

*MAO* monoamine oxidase, *SSRI* selective serotonin reuptake inhibitor

It is recommended, therefore, that diagnostic work-up includes assessment of TSH, free thyroxine (FT4), and creatinine levels to exclude secondary causes of hyperprolactinaemia. Many patients with hyperprolactinaemia have a predominance of high-molecular-weight prolactin (macroprolactinaemia) [202]. Most of these patients have unimpaired fertility and uneventful pregnancies, although they may present with some of the usual symptoms of hyperprolactinaemia such as galactorrhoea or menstrual disorders [202–204]. Patients with macroprolactinaemia generally do not require treatment, but the diagnosis is complicated by the fact that prolactin levels are highly variable and overlap with those found in patients with monomeric hyperprolactinaemia [205]. The diagnostic protocol for macroprolactinaemia should be included in the laboratory work-up for patients with hyperprolactinaemia, to spare patients from unnecessary hormonal or radiological investigations and treatments.

### Consequences of delayed diagnosis

Bone loss and vertebral fractures are the most common comorbidities of hyperprolactinaemia-mediated sex-steroid attenuation [206, 207]. In particular, spinal bone density is decreased by approximately 25 % in women with hyperprolactinaemia and may be permanent, although overt osteoporosis is rare [208]. Hyperprolactinaemia is an important cause of infertility in both women and men [185]. It can be reversed by appropriate treatment, for example with dopamine agonists [209, 210]. However, in some women with prolactinomas in whom hyperprolactinaemia has been corrected, two problems may potentially arise. First is the potential risk of the dopamine agonist to early fetal development, although reassuring data have been collected in pregnant women treated with bromocriptine and – in more limited cohorts – those treated with cabergoline [211]. Second, pregnancy itself may be detrimental because the increase in oestrogen levels may stimulate tumour growth [212, 213].

Although metabolic consequences of untreated hyperprolactinaemia can be expected, only limited data have been reported on the involvement of hyperprolactinaemia in the pathogenesis of obesity, glucose intolerance, and an impaired metabolic profile [214]. Importantly, treatment of prolactinoma has been shown to reduce the prevalence of metabolic syndrome and to improve the metabolic profile [215, 216].

## Pituitary thyrotroph adenomas

Pituitary adenomas that produce TSH (TSH-omas) accounted for 0.7 % of pituitary adenomas in the study by Tjörnstrand et al. giving a SIR of 0.03 per 100,000 [172]. Another Swedish study reported the national prevalence in 2010 as 2.8 per 1 million inhabitants [217]. These data support earlier epidemiological reports suggesting that thyrotroph adenomas account for between 0.5 % and 2.0 % of pituitary adenomas overall [218, 219]. Unlike incidences of the other secreting pituitary adenomas, that of thyrotroph adenomas is similar in women and men [218].

Patients with TSH-omas usually present with signs and symptoms typical of hyperthyroidism, and the presence of goitre is an almost constant symptom of pituitary thyrotroph adenoma [4]. The typical features include nervousness, irritability, increased perspiration, increased heart rate, hand tremors, anxiety, difficulty sleeping, muscular weakness, frequent diarrhoea, weight loss, and oligo-amenorrhoea. However, these features may be overshadowed by symptoms related to hypersecretion or deficiency of other pituitary hormones [4]. As reported in an overview by Beck-Peccoz et al. [4], dysfunction of the gonadal axis is less common than hyperthyroid features in patients with TSH-omas, but it is not rare. Menstrual disorders occur in approximately one-third of cases in females, mainly those with mixed TSH or prolactin-secreting adenomas. In males with TSH-secreting pituitary adenomas, central hypogonadism, delayed puberty, and decreased libido may manifest [4]. The predominant signs and symptoms of pituitary thyrotroph adenomas are related to expanding tumour mass, including hypopituitarism, headaches (in 20–25 % of patients), and visual field defects (in 50 % of patients) [4].

For biochemical diagnosis, European guidelines for the diagnosis of thyrotropin-secreting pituitary tumours recommend the measurement of circulating free tri-iodothyronine (FT3) and FT4 by using “two-step” methods (e.g. equilibrium dialysis and radioimmunoassay or adsorption chromatography and radioimmunoassay, with back-titration) in addition to TSH measurement for accurate differential diagnosis [220]. TSH-oma should be suspected if the patient presents with hyperthyroidism and high circulating levels of FT4 and FT3 and if TSH is not suppressed in thyrotropin-releasing hormone stimulation tests [220]. Serum TSH levels in patients with TSH-oma are mildly elevated or in the normal range [220]. In the context of elevated thyroid hormone levels, a normal TSH value excludes *a priori* peripheral thyroid disease (e.g. Graves’ disease) except when there is resistance to thyroid hormones [221]. Therefore, TSH measurement is mandatory in the diagnostic procedure in case of hyperthyroidism.

### Considerations for accurate diagnosis

Using the two-step methods suggested by Beck-Peccoz et al., other conditions that can result in detectable serum TSH and hyperthyroxinaemia (e.g. pregnancy, resistance to thyroid hormone [RTH], familial dysalbuminaemic hyperthyroxinaemia, and presence of T3 or T4 autoantibodies or circulating heterophilic antibodies) can be distinguished from hyperthyroidism secondary to secreting pituitary thyrotroph adenomas [4]. Differential diagnosis of pituitary thyrotroph adenomas and hyperthyroidism due to RTH syndrome can be made by using the following criteria: family history (signifies RTH not TSH-oma); pituitary lesions on imaging (signify TSH-oma); and germinal thyroid hormone receptor beta mutation (signifies RTH not TSH-oma) [4]. In cases with elevated levels of the alpha-subunit of pituitary glycoprotein hormone (a-GSU), elevated sex-hormone-binding globulin, and a high molar a-GSU:TSH ratio, TSH-oma rather than RTH can be suspected.

### Consequences of delayed diagnosis

Early diagnosis and proper treatment of TSH-omas can prevent the appearance of the signs and symptoms associated with mechanical compression of the adjacent structures by the expanding tumour mass (i.e. visual field defects, headache, and hypopituitarism) [4]. In addition, accurate diagnosis prevents improper thyroid ablation in those patients with central hyperthyroidism in whom the clinical manifestations of TSH-omas would not be prevented [220].

## Conclusions

Secreting pituitary adenomas are rare conditions that remain under-diagnosed. Diagnosis – particularly of acromegaly and Cushing’s disease – can be challenging because healthcare professionals not dedicated to pituitary disorders may lack awareness of these diseases and may therefore neglect the “red flags” that may suggest them. The typical physical features are slow to manifest, and because patients generally present with signs and symptoms that overlap with those of conditions commonly seen in primary care, diagnosis may be missed or delayed. A long duration of active disease is associated with an increased risk of comorbidities, reduced quality of life, and increased mortality. Increasing efforts to support the early diagnosis and treatment of these diseases is warranted, and the deleterious effects of secreting pituitary adenomas should not be overlooked.

## Abbreviations

ACTH: Adrenocorticotropic hormone
a-GSU: Alpha-subunit of pituitary glycoprotein hormone
BMD: Bone mineral density
FT3: Free tri-iodothyronine
FT4: Free thyroxine
GH: Growth hormone
GP: General practitioner
IGF-1: Insulin-like growth factor 1
OGTT: Oral glucose tolerance test
RTH: Resistance to thyroid hormone
SIR: Standardized incidence rate
TSH: Thyroid-stimulating hormone
UFC: Urinary free cortisol
